# Supplementary material for: A four-DNA methylation biomarker is a superior predictor of survival of patients with cutaneous melanoma
Source: eLife. 2019 Jun 6;8:e44310. doi: 10.7554/eLife.44310 (PMC6553943; doi:10.7554/eLife.44310)
Supplement: Supplementary file 1. [file elife-44310-supp1.docx]

**Supplementary file 1.** Four significantly survival-related methylation sites in training dataset.

| **Probe ID** | **Chromosomal location** | **Gene symbol** | **CGI coordinate** | **Feature type** | ***P* value^a^** | **Coef.^b^** | ***P* value^b^** |
| --- | --- | --- | --- | --- | --- | --- | --- |
| cg06778853 | chr1: 6612560–6612561 | *KLHL21* | chr1:6612726–6615681 | N_Shore | 1.71E-04 | –1.912 | 1.06E-03 |
|  |  |  |  |  |  |  |  |
| cg24670442 | chr1: 89270795–89270796 | *GBP5* | chr1:89524251–89525427 | NA | 4.02E-04 | 4.262 | 1.17E-02 |
|  |  |  |  |  |  |  |  |
| cg18456782 | chr15: 27806569–27806570 | *OCA2* | chr15:27806392–27806644 | Island | 5.64E-04 | 1.229 | 1.43E-04 |
|  |  |  |  |  |  |  |  |
| cg26263675 | chr17: 74747052–74747053 | *RAB37* | chr17:74748289–74749813 | N_Shore | 2.90E-05 | –2.108 | 4.08E-05 |
|  |  |  |  |  |  |  |  |
| ^a^. adjusted *P* value with Benjamini and Hochberg False Discovery Rate correction in univariate Cox regression analysis; | | | | | | | |
| ^b^. in multivariate Cox regression analysis; | | | | | | | |
