# Supplementary material for: A four-DNA methylation biomarker is a superior predictor of survival of patients with cutaneous melanoma
Source: eLife. 2019 Jun 6;8:e44310. doi: 10.7554/eLife.44310 (PMC6553943; doi:10.7554/eLife.44310)
Supplement: Supplementary file 2. [file elife-44310-supp2.docx]

**Supplementary file 2.** The ROC results of four-DNA methylation signature and other known biomarkers.

| **Signature** | **AUC** | **95% CI of AUC** | ***P* value^a^** | **Type** | ***P* value^b^** | **Ref** |
| --- | --- | --- | --- | --- | --- | --- |
|  |  |  |  |  |  |  |
| Four-DNA methylation | 0.754 | 0.66–0.85 | < 0.001 | Methylation |  | This study |
| Four-lncRNA | 0.634 | 0.52–0.75 | 0.023 | LncRNA | 0.061 | (Chen et al., 2017) |
| *BANCR* | 0.597 | 0.48–0.71 | 0.102 | LncRNA | 0.020 | (Li et al., 2014) |
| *H19* | 0.501 | 0.38–0.62 | 0.989 | LncRNA | 0.001 | (Shi et al., 2018) |
| *CD24* | 0.520 | 0.40–0.63 | 0.736 | Protein coding | 0.001 | (Tang et al., 2014) |
| *CD74* | 0.604 | 0.49–0.72 | 0.080 | Protein coding | 0.026 | (Ekmekcioglu et al., 2016) |
| *CTLA-4* | 0.622 | 0.51–0.74 | 0.040 | Protein coding | 0.042 | (Goltz et al., 2018) |
| *DCTN1* | 0.628 | 0.52–0.74 | 0.030 | Protein coding | 0.045 | (Wang et al., 2018) |
| *DCTN2* | 0.612 | 0.50–0.72 | 0.059 | Protein coding | 0.029 | (Wang et al., 2018) |
| *DCTN5* | 0.534 | 0.42–0.65 | 0.566 | Protein coding | 0.002 | (Wang et al., 2018) |
| *EGFR* | 0.545 | 0.43–0.66 | 0.447 | Protein coding | 0.003 | (Katunaric et al., 2014) |
| *eIF4E* | 0.557 | 0.44–0.67 | 0.332 | Protein coding | 0.005 | (Khosravi et al., 2015) |
| *Fbw7* | 0.599 | 0.49–0.71 | 0.093 | Protein coding | 0.021 | (Cheng et al., 2013) |
| *GEF-H1* | 0.508 | 0.39–0.62 | 0.892 | Protein coding | 0.001 | (Shi et al., 2016) |
| *KAI1* | 0.513 | 0.40–0.60 | 0.824 | Protein coding | 0.001 | (Zhang et al., 2015) |
| *MCM3* | 0.523 | 0.41–0.64 | 0.698 | Protein coding | 0.001 | (Nodin et al., 2012) |
| *MITF* | 0.538 | 0.42–0.65 | 0.523 | Protein coding | 0.002 | (Garraway et al., 2005) |
| *MMP2* | 0.564 | 0.45–0.68 | 0.278 | Protein coding | 0.007 | (Rotte et al., 2012) |
| *P27* | 0.502 | 0.38–0.62 | 0.977 | Protein coding | 0.001 | (Zhang et al., 2015) |
| *RAGE* | 0.571 | 0.46–0.69 | 0.231 | Protein coding | 0.009 | (Wagner et al., 2015) |
| *SART3* | 0.531 | 0.42–0.65 | 0.600 | Protein coding | 0.002 | (Timani et al., 2018) |
| *SOX9* | 0.542 | 0.43–0.66 | 0.477 | Protein coding | 0.003 | (Cheng et al., 2015) |
| *Tip60* | 0.520 | 0.40–0.64 | 0.741 | Protein coding | 0.001 | (Chen et al., 2012) |
| *TRPM1* | 0.538 | 0.42–0.65 | 0.523 | Protein coding | 0.002 | (Brozyna et al., 2017) |
| 17-DNA Methylation | 0.619 | 0.53–0.77 | 0.044 | Methylation | 0.035 | (Sigalotti et al., 2012) |
| *CTLA-4* | 0.516 | 0.40–0.63 | 0.058 | Methylation | 0.002 | (Goltz et al., 2018) |
| *ER-α* | 0.700 | 0.48–0.70 | 0.001 | Methylation | 0.227 | (Mori et al., 2006) |
| *HOXD9* | 0.592 | 0.48–0.70 | 0.116 | Methylation | 0.016 | (Marzese et al., 2014) |
| *LINE-1* | 0.617 | 0.51–0.73 | 0.048 | Methylation | 0.068 | (Sigalotti et al., 2011) |
| *LKB1* | 0.634 | 0.52–0.75 | 0.023 | Methylation | 0.114 | (Zhang et al., 2017) |
| *MGMT* | 0.592 | 0.59–0.80 | 0.133 | Methylation | 0.018 | (Cesinaro et al., 2012) |
| *PTEN* | 0.605 | 0.49–0.72 | 0.074 | Methylation | 0.025 | (Roh et al., 2016) |
| *TNFRSF10D* | 0.651 | 0.48–0.71 | 0.016 | Methylation | 0.092 | (Ratzinger et al., 2014) |
| ^a^. in ROC analysis; | | | | | | |
| ^b^. in the statistical comparison (Z-test) between AUC value of corresponding signature and the five-DNA methylation signature. | | | | | | |

**References**

Brozyna, A.A., Guo, H., Yang, S.E., Cornelius, L., Linette, G., Murphy, M., Sheehan, C., Ross, J., Slominski, A., and Carlson, J.A. (2017). TRPM1 (melastatin) expression is an independent predictor of overall survival in clinical AJCC stage I and II melanoma patients. Journal of cutaneous pathology *44*, 328-337.

Cesinaro, A.M., Sartori, G., Migaldi, M., Schirosi, L., Pellacani, G., Collina, G., and Maiorana, A. (2012). Prognostic significance of MGMT gene promoter methylation in differently treated metastatic melanomas. Pathology *44*, 313-317.

Chen, G., Cheng, Y., Tang, Y., Martinka, M., and Li, G. (2012). Role of Tip60 in human melanoma cell migration, metastasis, and patient survival. The Journal of investigative dermatology *132*, 2632-2641.

Chen, X., Guo, W., Xu, X.J., Su, F., Wang, Y., Zhang, Y., Wang, Q., and Zhu, L. (2017). Melanoma long non-coding RNA signature predicts prognostic survival and directs clinical risk-specific treatments. Journal of dermatological science *85*, 226-234.

Cheng, P.F., Shakhova, O., Widmer, D.S., Eichhoff, O.M., Zingg, D., Frommel, S.C., Belloni, B., Raaijmakers, M.I., Goldinger, S.M., Santoro, R.*, et al.* (2015). Methylation-dependent SOX9 expression mediates invasion in human melanoma cells and is a negative prognostic factor in advanced melanoma. Genome biology *16*, 42.

Cheng, Y., Chen, G., Martinka, M., Ho, V., and Li, G. (2013). Prognostic significance of Fbw7 in human melanoma and its role in cell migration. The Journal of investigative dermatology *133*, 1794-1802.

Ekmekcioglu, S., Davies, M.A., Tanese, K., Roszik, J., Shin-Sim, M., Bassett, R.L., Jr., Milton, D.R., Woodman, S.E., Prieto, V.G., Gershenwald, J.E.*, et al.* (2016). Inflammatory Marker Testing Identifies CD74 Expression in Melanoma Tumor Cells, and Its Expression Associates with Favorable Survival for Stage III Melanoma. Clinical cancer research : an official journal of the American Association for Cancer Research *22*, 3016-3024.

Garraway, L.A., Widlund, H.R., Rubin, M.A., Getz, G., Berger, A.J., Ramaswamy, S., Beroukhim, R., Milner, D.A., Granter, S.R., Du, J.*, et al.* (2005). Integrative genomic analyses identify MITF as a lineage survival oncogene amplified in malignant melanoma. Nature *436*, 117-122.

Goltz, D., Gevensleben, H., Vogt, T.J., Dietrich, J., Golletz, C., Bootz, F., Kristiansen, G., Landsberg, J., and Dietrich, D. (2018). CTLA4 methylation predicts response to anti-PD-1 and anti-CTLA-4 immunotherapy in melanoma patients. JCI insight *3*.

Katunaric, M., Jurisic, D., Petkovic, M., Grahovac, M., Grahovac, B., and Zamolo, G. (2014). EGFR and cyclin D1 in nodular melanoma: correlation with pathohistological parameters and overall survival. Melanoma research *24*, 584-591.

Khosravi, S., Tam, K.J., Ardekani, G.S., Martinka, M., McElwee, K.J., and Ong, C.J. (2015). eIF4E is an adverse prognostic marker of melanoma patient survival by increasing melanoma cell invasion. The Journal of investigative dermatology *135*, 1358-1367.

Li, R., Zhang, L., Jia, L., Duan, Y., Li, Y., Bao, L., and Sha, N. (2014). Long non-coding RNA BANCR promotes proliferation in malignant melanoma by regulating MAPK pathway activation. PloS one *9*, e100893.

Marzese, D.M., Scolyer, R.A., Huynh, J.L., Huang, S.K., Hirose, H., Chong, K.K., Kiyohara, E., Wang, J., Kawas, N.P., Donovan, N.C.*, et al.* (2014). Epigenome-wide DNA methylation landscape of melanoma progression to brain metastasis reveals aberrations on homeobox D cluster associated with prognosis. Human molecular genetics *23*, 226-238.

Mori, T., Martinez, S.R., O'Day, S.J., Morton, D.L., Umetani, N., Kitago, M., Tanemura, A., Nguyen, S.L., Tran, A.N., Wang, H.J., and Hoon, D.S. (2006). Estrogen receptor-alpha methylation predicts melanoma progression. Cancer research *66*, 6692-6698.

Nodin, B., Fridberg, M., Jonsson, L., Bergman, J., Uhlen, M., and Jirstrom, K. (2012). High MCM3 expression is an independent biomarker of poor prognosis and correlates with reduced RBM3 expression in a prospective cohort of malignant melanoma. Diagnostic pathology *7*, 82.

Ratzinger, G., Mitteregger, S., Wolf, B., Berger, R., Zelger, B., Weinlich, G., Fritsch, P., Goebel, G., and Fiegl, H. (2014). Association of TNFRSF10D DNA-methylation with the survival of melanoma patients. International journal of molecular sciences *15*, 11984-11995.

Roh, M.R., Gupta, S., Park, K.H., Chung, K.Y., Lauss, M., Flaherty, K.T., Jonsson, G., Rha, S.Y., and Tsao, H. (2016). Promoter Methylation of PTEN Is a Significant Prognostic Factor in Melanoma Survival. The Journal of investigative dermatology *136*, 1002-1011.

Rotte, A., Martinka, M., and Li, G. (2012). MMP2 expression is a prognostic marker for primary melanoma patients. Cellular oncology *35*, 207-216.

Shi, G., Li, H., Gao, F., and Tan, Q. (2018). lncRNA H19 predicts poor prognosis in patients with melanoma and regulates cell growth, invasion, migration and epithelial-mesenchymal transition in melanoma cells. OncoTargets and therapy *11*, 3583-3595.

Shi, J., Guo, B., Zhang, Y., Hui, Q., Chang, P., and Tao, K. (2016). Guanine nucleotide exchange factor H1 can be a new biomarker of melanoma. Biologics : targets & therapy *10*, 89-98.

Sigalotti, L., Covre, A., Fratta, E., Parisi, G., Sonego, P., Colizzi, F., Coral, S., Massarut, S., Kirkwood, J.M., and Maio, M. (2012). Whole genome methylation profiles as independent markers of survival in stage IIIC melanoma patients. Journal of translational medicine *10*, 185.

Sigalotti, L., Fratta, E., Bidoli, E., Covre, A., Parisi, G., Colizzi, F., Coral, S., Massarut, S., Kirkwood, J.M., and Maio, M. (2011). Methylation levels of the "long interspersed nucleotide element-1" repetitive sequences predict survival of melanoma patients. Journal of translational medicine *9*, 78.

Tang, M.R., Wang, Y.X., Guo, S., Han, S.Y., Li, H.H., and Jin, S.F. (2014). CD24 expression predicts poor prognosis for patients with cutaneous malignant melanoma. International journal of clinical and experimental medicine *7*, 4337-4341.

Timani, K.A., Gyorffy, B., Liu, Y., Mohammad, K.S., and He, J.J. (2018). Tip110/SART3 regulates IL-8 expression and predicts the clinical outcomes in melanoma. Molecular cancer *17*, 124.

Wagner, N.B., Weide, B., Reith, M., Tarnanidis, K., Kehrel, C., Lichtenberger, R., Pflugfelder, A., Herpel, E., Eubel, J., Ikenberg, K.*, et al.* (2015). Diminished levels of the soluble form of RAGE are related to poor survival in malignant melanoma. International journal of cancer. Journal international du cancer *137*, 2607-2617.

Wang, Q., Wang, X., Liang, Q., Wang, S., Liao, X., Li, D., and Pan, F. (2018). Prognostic Value of Dynactin mRNA Expression in Cutaneous Melanoma. Medical science monitor : international medical journal of experimental and clinical research *24*, 3752-3763.

Zhang, G., Cheng, Y., Chen, G., Tang, Y., Ardekani, G., Rotte, A., Martinka, M., McElwee, K., Xu, X., Wang, Q., and Zhou, Y. (2015). Loss of tumor suppressors KAI1 and p27 identifies a unique subgroup of primary melanoma patients with poor prognosis. Oncotarget *6*, 23026-23035.

Zhang, W., Li, X., Song, G., and Luo, D. (2017). Prognostic significance of LKB1 promoter methylation in cutaneous malignant melanoma. Oncology letters *14*, 2075-2080.
