# Supplementary material for: A four-DNA methylation biomarker is a superior predictor of survival of patients with cutaneous melanoma
Source: eLife. 2019 Jun 6;8:e44310. doi: 10.7554/eLife.44310 (PMC6553943; doi:10.7554/eLife.44310)
Supplement: Supplementary file 3. [file elife-44310-supp3.docx]

**Supplementary file 3.** The correlation raw P values and adjusted P values with the Bonferroni correction between our 4-meth signature and other known signatures.

| **P-value^a^** | **4-meth** | **PD-1** | **PD-L1** | **PD-L2** | **CTLA4** | **TMB** | **MeTIL** | **GBP5** |
| --- | --- | --- | --- | --- | --- | --- | --- | --- |
| **4-meth** |  | 1.67E-28 | 1.92E-14 | 1.48E-12 | 0.00502 | 0.781 | 9.04E-21 | 1.60E-49 |
| **PD-1** | 1.17E-27 |  | 3.22E-21 | 1.26E-22 | 4.00E-06 | 0.105 | 3.46E-20 | 9.70E-64 |
| **PD-L1** | 1.34E-13 | 2.25E-20 |  | 1.41E-89 | 0.018 | 0.748 | 2.85E-10 | 1.58E-19 |
| **PD-L2** | 1.04E-11 | 8.82E-22 | 9.87E-89 |  | 0.012 | 0.179 | 3.14E-10 | 1.07E-22 |
| **CTLA4** | 0.0351 | 2.80E-05 | 0.126 | 0.084 |  | 0.354 | 0.233 | 2.30E-04 |
| **TMB** | 5.467 | 0.735 | 5.236 | 1.253 | 2.478 |  | 0.461 | 0.083 |
| **MeTIL** | 6.33E-20 | 2.42E-19 | 2.00E-09 | 2.20E-09 | 1.631 | 3.227 |  | 3.07E-17 |
| **GBP5** | 1.12E-48 | 6.79E-63 | 1.11E-18 | 7.49E-22 | 0.0016 | 0.581 | 2.15E-16 |  |
| a.  Upper triangle were raw P values, and lower triangle were adjusted P value with the Bonferroni correction. | | | | | | | | |
